# Real-Time Semantics-Driven Infrared and Visible Image Fusion Network

**DOI:** 10.3390/s23136113

**Published:** 2023-07-03

**Authors:** Binhao Zheng, Tieming Xiang, Minghuang Lin, Silin Cheng, Pengquan Zhang

**Affiliations:** School of Electronic Engineering, Hangzhou Dianzi University, Hangzhou 310018, China

**Keywords:** semantics-driven, image fusion, convolution neural network

## Abstract

This paper proposes a real-time semantics-driven infrared and visible image fusion framework (RSDFusion). A novel semantics-driven image fusion strategy is introduced in image fusion to maximize the retention of significant information of the source image in the fusion image. First, a semantically segmented image of the source image is obtained using a pre-trained semantic segmentation model. Second, masks of significant targets are obtained from the semantically segmented image, and these masks are used to separate the targets in the source and fusion images. Finally, the local semantic loss of the separation target is designed and combined with the overall structural similarity loss of the image to instruct the network to extract appropriate features to reconstruct the fusion image. Experimental results show that the RSDFusion proposed in this paper outperformed other comparative methods on both subjective and objective evaluation of public datasets and that the main target of the source image is better preserved in the fusion image.

## 1. Introduction

Due to technical limitations of hardware devices or optical imaging, images captured with a single type of sensor and recording device can only capture partial information and cannot effectively and comprehensively describe the scene being imaged [1]. For example, visible light images typically contain rich texture detail information but are susceptible to extreme environments, occlusions, etc., which can cause targets in the scene to be lost. Infrared sensors capture the thermal radiation emitted by objects and are effective in detecting prominent targets such as pedestrians and vehicles but lack a textural description of the scene [2]. As an important branch of image processing, image fusion plays an important role in this regard by effectively integrating complementary information between images captured by different sensors and recording devices and generating images that approximate the fused scene by performing reconstruction from multiple modal samples. Fusion images have complementary properties and better scene representation than the source images, enabling effective applications such as target recognition [3], clinical diagnosis [4], semantic segmentation [5], remote sensing monitoring [6], and applications in other areas.

Infrared and visible images have become a popular means for image fusion due to their powerful complementary information. In recent years, many infrared and visible image fusion algorithms have been proposed, which broadly fall into two categories: traditional algorithms and deep learning-based algorithms. Traditional image fusion algorithms mainly include methods based on multiscale transform [7,8], methods based on sparse representation [9,10], methods based on low-rank representation [11], methods based on subspace [12], and methods based on hybrid techniques [13]. Although the existing traditional image fusion methods achieve good fusion results in most cases, problems still need to be solved. First, traditional manually designed fusion strategies cannot be adapted to complex fusion scenes, which limits their fusion results. Second, traditional methods do not take into account the inherent differences between infrared and visible images, and it is difficult to extract the unique information between different modal images using the same method. With the introduction of deep learning in image processing in recent years, several infrared and visible image fusion techniques based on deep learning have quickly evolved. Depending on the network architecture used, three primary categories of deep learning-based image fusion techniques exist: auto-encoder (AE)-based frameworks [14,15], convolutional neural network (CNN)-based frameworks [16,17,18], and generative adversarial network (GAN)-based frameworks [19,20].

Current image fusion algorithms based on deep learning can effectively integrate infrared and visible images, but there are still several issues that need to be addressed. First, existing algorithms tend to use global fusion to process source image information, using the same processing scheme for each local target in the source image, without considering the importance of each target in the source image under different tasks, resulting in mediocre results in the local fusion of fusion images. Currently, some studies have constrained the network to perform local fusion by introducing pixel saliency, but pixel saliency does not allow efficient detection of the target objects in an image to be achieved. Second, the loss functions of existing image fusion networks mainly focus on the overall structural loss of the image and global pixel loss. Structural loss mainly refers to the loss of detail texture between the source image and the fusion image, while global pixel loss mainly refers to the loss of pixel size between the source image and the fusion image. However, neither of these two loss types can fully reflect the loss of pixels on locally important targets of the image.

To address the above issues, this paper introduces semantic segmentation into the image fusion task and proposes a semantics-driven framework for real-time infrared and visible image fusion. Specifically, we add the corresponding infrared and visible semantically segmented images to the original infrared and visible dataset to extract the target mask. After generating fusion images, the main semantic targets in the source and fusion images are extracted using the target mask, and the loss of the source and fusion images on the main semantic targets is calculated to constrain the fusion images to maximize the retention of local target features in the source images. In addition, this paper also designs semantic loss based on local target objects that can accurately reflect the pixel loss of the fusion image on local target objects.

The main contributions of this paper can be summarized as follows:

1. This paper proposes a real-time semantics-driven framework for infrared and visible image fusion that makes full use of semantic information to have the fusion images retain clearer semantic targets;
2. This paper proposes local target content loss that guides the fusion network to locally fuse the same targets extracted from infrared and visible images, effectively improving the local target quality of the fusion images;
3. Experimental results show that the proposed algorithm outperforms existing popular fusion algorithms in both subjective visualization and objective evaluation.

The rest of the paper is structured as follows: Section 2 briefly describes the work related to deep learning-based image fusion methods and semantic segmentation. Section 3 provides a specific description of the proposed fusion method. Section 4 presents comparative validation experiments. Section 5 summarizes the work performed in this thesis.

## 2. Related Work

This section reviews the available popular image fusion algorithms and provides a brief introduction to semantic segmentation.

### 2.1. Deep Learning-Based Image Fusion Methods

With deep learning techniques gaining ground in image processing, deep learning-based methods have been widely used in image fusion tasks. Among them, AE-based frameworks mainly pre-train a self-encoder on some large datasets to achieve feature extraction and image reconstruction and then integrate the features to achieve image fusion using some hand-designed fusion strategies. For example, Li et al. proposed the DenseFuse method [14], which has three parts: encoder, feature fusion layer, and decoder. In the encoder layer, dense blocks are introduced to obtain deeper features while effectively improving the transfer of features from the previous layers. In the fusion layer, element-by-element addition and L1 parametric strategy are used for feature fusion. In addition, they proposed a multiscale fusion method, NestFuse [15], which uses a multiscale encoder–decoder architecture and nested linking modules to enhance the deeper feature extraction capability and uses a spatial/channel attention model in the fusion layer instead of weighting and L1 parametric strategies. Although the above approaches achieve good performance, their handcrafted fusion strategies are difficult to adapt to complex fusion scenarios.

CNN-based frameworks are end-to-end image fusion frameworks that can effectively avoid the disadvantages of handcrafted fusion strategies. Liu et al. first proposed a CNN-based fusion method using 16 × 16 paired image blocks to train the network to build decision maps [16]. However, the authors pointed out that the trained network was only suited to the multi-focus image fusion task but not other fusion tasks. Zhang et al. proposed a general fusion framework, IFCNN [17], which first extracts salient features from the source image using two convolutional layers to determine the image class; then, it selects a specific fusion strategy to fuse the features according to the input image type; finally, it generates a fused image. Lin et al. proposed a semantics-aware image fusion network, SeAFusion [18]. It effectively improves the performance of fused images in advanced vision tasks by adding a semantic segmentation module after the image fusion module and feeding the semantic information back to the image fusion module using semantic loss.

GAN-based frameworks are also end-to-end image fusion frameworks that avoid hand-designed fusion strategies using adversarial learning between generators and discriminators. Ma et al. first proposed FusionGAN [19]. This framework constrains the fusion image to obtain more information from the source image by establishing an adversarial game mechanism between the generator and the discriminator. However, the fusion images generated using this framework have poor retention of infrared salient targets. Therefore, Ma et al. introduced a dual discriminator fusion framework, DDcGAN [20]. This framework has two discriminators that are separately evaluated for infrared and visible images. Subsequently, Li et al. presented AttentionGAN [21], which incorporates a multiscale attention mechanism into the GAN architecture to improve the retention of significant features in the source image by enhancing the attention region with the generator and discriminator.

In general, both traditional and deep learning-based approaches emphasize the quality and metrics of the fusion image as a whole while ignoring the importance of the main target object. In practice, the information of interest is what needs to be observed, while the information of disinterest is what can be relatively ignored. Therefore, the targets in the image are segmented and assigned appropriate weights according to the needs of different real tasks. Semantic segmentation is a quality technique for dividing targets in each region of the image.

### 2.2. Semantic Segmentation

Semantic segmentation is one of the fundamental tasks in computer vision today, and its main function is to let the computer segment an object based on the semantics in the image and determine what and where the object is at the pixel level. In recent years, semantic segmentation methods based on deep learning have attracted much attention due to their excellent performance and powerful generalization capabilities. First, Long et al. proposed a semantic segmentation method, FCN [22], which innovatively applies deep learning methods to semantic segmentation. FCN replaces the fully connected layers of the network with convolutional layers to achieve semantic segmentation at arbitrary resolutions. Olaf et al. proposed the UNet architecture [23] based on the self-encoder architecture, which was the first network applied to medical image segmentation, and this network preserves the high-level semantic information and low-level positional information of the source image using jumping connection. Chen et al. used null convolution instead of traditional convolution to increase the density of features while maintaining the spatial resolution [24]. Xie et al. proposed SegFormer, a segmentation algorithm based on a transformer with a multilayer perceptron [25]. Since semantic segmentation can classify images at the pixel level based on their semantic features, it can be an effective method to classify different target objects in an image. There are some practical schemes that combine the image fusion task and the semantic segmentation task. For example, Zhou et al. introduced image fusion using semantic segmentation using a mask with semantic information to divide the source image into foreground and background regions and using a generative adversarial model to fuse the infrared foreground with the visible foreground, and the infrared background with the visible background [26]. Tang et al. proposed SeAFusion, which concatenates a semantic segmentation module after the fusion module. This approach aims to use semantic loss to guide the integration of high-level semantic information feedback into the fusion module, thus improving the performance of fused images in advanced vision tasks. However, we believe that it is more important to select different salient target objects in the source image to be enhanced according to different scenes under the same computational power, while the remaining objects can be relatively ignored. For this purpose, we propose a new semantics-driven image fusion algorithm, RSDFusion.

## 3. Proposed Method

This section introduces the proposed semantics-driven infrared and visible light image fusion framework, RSDFusion.

### 3.1. General Framework

Image fusion is a technique for extracting and integrating important information from source images. The key to this technique is how to select significant information in the source images. In different scenarios, different target information has different importance. For example, in autonomous driving, information on people, vehicles, and roads is more important than other information. In other words, the targets in the source image need to be weighted differently according to different scenes. In this context, semantic segmentation techniques can help to select the important targets in the source image. Therefore, we designed a semantics-driven image fusion network using semantic segmentation, so that the fusion image can more effectively preserve the semantic information of important targets in the source image according to the needs.

**Figure 1:**
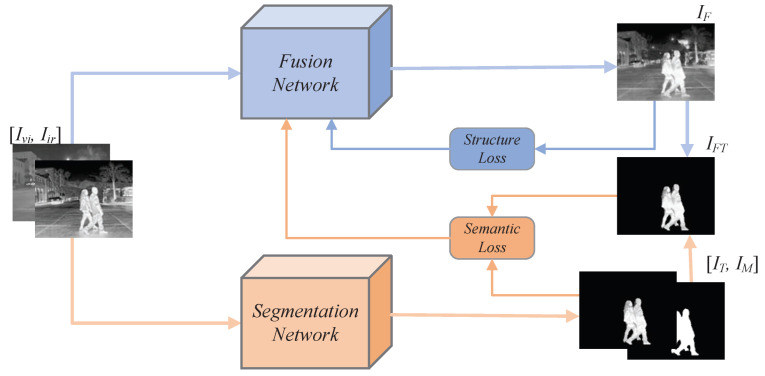
The general framework of the proposed RSDFusion.

The general framework of our proposed RSDFusion is shown in Figure 1. In a first step, the infrared image ($I_{ir}$) and visible image ($I_{vi}$) are input into the fusion network (F(·)) to obtain a fusion image ($I_{F}$), which is expressed as

(1) $$I_{F}=F\left(I_{vi},I_{ir}\right)$$

Meanwhile, $I_{ir}$ and $I_{vi}$ are fed into the semantic segmentation network (S(·)) to generate the mask ($I_{M}$). The source image target ($I_{T}$) is obtained with the weight function (W(·)) and the mask, which can be expressed as

(2) $$I_{M}=S\left(I_{vi},I_{ir}\right)$$

(3) $$I_{T}=I_{M}\cdot W\left(I_{vi},I_{ir}\right)$$

Then, the fusion image target ($I_{FT}$) is obtained by masking and fusing the images, which can be expressed as

(4) $$I_{FT}=I_{M}\cdot I_{F}$$

Finally, the structural loss function (ST(·)) is used to calculate the structural loss of the fusion and source images, and the semantic loss function (SE(·)) is used to calculate the semantic loss of the fusion image and source image targets; after summing structural loss and semantic loss to obtain the total loss ($L_{total}$), the total loss is fed back into the network using the backpropagation method to update the network parameters. The $L_{total}$ function is defined as follows:

(5) $$L_{total}=ST\left(I_{F},I_{vi},I_{ir}\right)+SE\left(I_{FT},I_{T}\right)$$

### 3.2. Network Architecture

**Figure 2:**
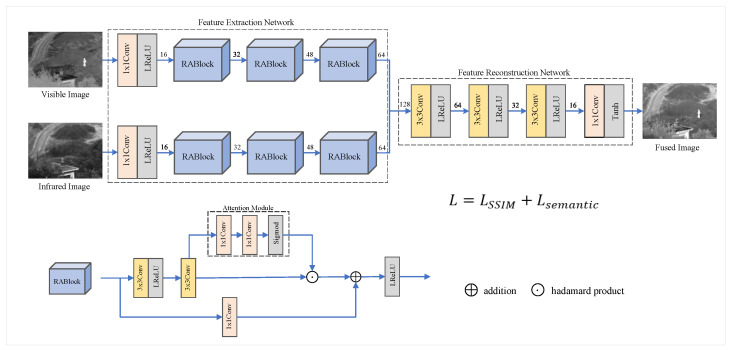
The network architecture of the proposed RSDFusion.

The architecture of RSDFusion is shown in Figure 2. It has two main parts: a feature extraction network and a feature reconstruction network.

The feature extraction network contains two separate feature extractors. Each feature extractor contains a 1 × 1 convolutional block and three RABlocks tuned according to ResBlock [27]. Each RABlock consists of two 3 × 3 convolutional blocks with BN layers, a spatial attention module, and a hopping 1 × 1 convolutional layer. The spatial attention block contains two 1 × 1 convolution layers and a sigmoid function. RABlock improves the feature extraction network and the ability of deep learning to focus on essential features while reducing gradient disappearance or explosion. The activation function in the network is the leakage modified linear unit (LReLU). This function can help to speed up network training while solving the problem of non-learning neurons. In addition, due to the differences between the infrared and visible images, the two extractors have identical network structures and independent convolutional parameters.

The feature reconstruction network contains three 3 × 3 convolutional blocks and one 1 × 1 convolutional block. Among them, the activation function used after the 1 × 1 convolutional block is the hyperbolic tangent function (Tanh). This function ensures that the fusion image has the same range of variation as the input image.

### 3.3. Semantic Segmentation Module

**Figure 3:**
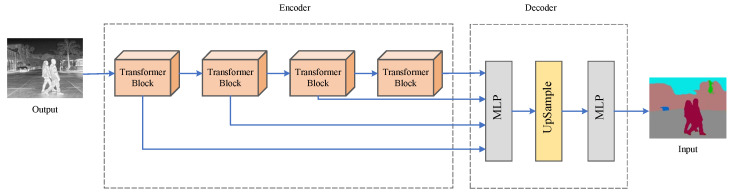
The specific architecture of SegFormer.

We used the semantic segmentation network SegFormer to semantically segment the infrared and visible dataset RoadScene and manually correct the segmented images to finally obtain a new infrared and visible dataset, RRS (RoadScene-Seg), with semantically segmented images. The SegFormer network is a lightweight segmentation network based on a transformer and multilayer perceptron that has the advantages of few parameters, fast training, and being powerful. The structure of the SegFormer network is shown in Figure 3. It uses an auto-encoder structure: The encoder consists of four transformer blocks that can output features at different scales. The decoder uses a lightweight multilayer perceptron (MLP) to aggregate multiscale features and the UpSample layer to recover the original resolution. The training dataset of this network is ADE20K [28], which covers a wide range of scenes and object classes. After the semantic segmentation of the image, each target object of the semantic image can be easily extracted by following the palette in the ADE20K dataset.

A total of 1908 images are included in the RSS dataset. Each image set contains an infrared image, a visible image, an infrared semantically segmented image, and a visible semantically segmented image. The segmented source image contains a large number of semantic objects, but not all of them are necessary. Therefore, semantics-based image fusion is concerned with how to select the necessary semantic target objects. In this paper, we argue that the targets of the source image should be suitable to the work to be performed. For different usage scenarios, the appropriate key target objects should be selected. For example, the segmentation of people, cars, and roads is crucial in autonomous driving. Therefore, in this image fusion process, the semantic targets of people, cars, and roads are given higher priority and are the important semantic target objects to be retained in the final fusion image. In addition, the semantic segmentation images of infrared and visible images are very different, and some semantic objects only exist in infrared or visible images. Therefore, to avoid semantic dropouts of important targets from the source images, we use masks for people and cars in infrared images, masks for roads and plants in visible photos, and masks for the sky in infrared and visible images.

### 3.4. Loss Function

To better preserve the source image’s overall structure and enhance the fusion image’s semantic information, we propose a new loss function. It is mainly divided into global structural loss ($L_{SSIM}$) and local semantic object loss ($L_{semantic}$). Our loss function is defined as follows:

(6) $$L=\alpha L_{SSIM}+\beta L_{\mathrm{semantic}\;}$$

where $L_{SSIM}$ mainly constrains the global structural features in the fusion image, while $L_{semantic}$ constrains the fusion image to retain more detailed features and semantic information of the source image target. α and β are used as adjustment factors to balance the global structural loss and partial semantic loss.

The global structural loss ($L_{SSIM}$) is obtained by calculating the sum of the structural loss between the fusion image and the infrared and visible images as follows:

(7) $$L_{SSIM}=\omega_{1}\left(1-\mathrm{SSIM}\left(I_{f},I_{ir}\right)\right)+\omega_{2}\left(1-\mathrm{SSIM}\left(I_{f},I_{vi}\right)\right)$$

where $I_{f}$ is the fusion image; $I_{ir}$ is the infrared image; $I_{vi}$ is the visible image; and $\omega_{1}$ and $\omega_{2}$ are the structural loss coefficients, which are mainly used to control the degree of influence of infrared and visible structural features on the fusion image during the fusion process. $\omega_{1}$ and $\omega_{2}$ have values in the range [0, 1], and $\omega_{1}$ + $\omega_{2}$ = 1. The SSIM [29] function considers three elements of the image: luminance loss, contrast loss, and texture loss. The SSIM formula is as follows:

(8) $$SSIM\left(x,y\right)=l\left(x,y\right)\cdot c\left(x,y\right)\cdot s\left(x,y\right)$$

where l(x,y) represents the loss of global brightness of the fusion image, c(x,y) represents the loss of global contrast of the fusion image, and s(x,y) represents the loss of global structural similarity between fusion image and source image.

The local semantic target loss ($L_{semantic}$) can drive the locally important targets of the fusion image toward the source image. Infrared and visible images are segmented with a semantic segmentation model, and the semantic objects in the segmented images are extracted using a mask. However, a large number of semantic objects are extracted from the source images. For the application scenario of the training set used, we set the main targets as people, vehicles, sky, roads, and plants. The local semantic object loss function is defined as follows:

(9) $$L_{semantic}=\lambda_{1}L_{person}+\lambda_{2}L_{car}+\lambda_{3}L_{sky}+\lambda_{4}L_{load}+\lambda_{5}L_{green}$$

where the local semantic loss of the person is defined as

(10) $$L_{person}=\frac{1}{N}{∥F_{person}-\left(\omega_{3}*{IR}_{person}+\omega_{4}*{VIS}_{person}\right)∥}_{2}^{2}$$

where $F_{person}$ is the person pixel in the fusion image, $IR_{person}$ is the person pixel in the infrared image, $VIS_{person}$ is the person pixel in the visible image, ${∥\cdot ∥}_{2}$ represents the $l_{2}$-norm, N represents the number of pixels within the mask that have a value of 1, and $\omega_{3}$ and $\omega_{4}$ denote the person pixel significance coefficients in the infrared and visible images. The range of the latter is [0, 1], and $\omega_{3}+\omega_{4}=1$.

## 4. Experimental Validation

In this section, we first present the experimental setup of this work. Then, we contrast the proposed method with nine other representative methods on public datasets. Finally, we further validate the efficiency of the proposed method with ablation and efficiency experiments.

### 4.1. Experimental Configuration

(1) Datasets: We performed quantitative and qualitative experiments on our algorithms using the RoadScene and TNO datasets. The RoadScene dataset is an infrared and visible light dataset for autonomous driving containing 221 image pairs including people, vehicles, and roads. The TNO dataset is currently the most classic dataset for infrared and visible light image fusion tasks, and it consists of 60 image pairs from different military scenes taken with cameras at different wavelengths.

(2) Evaluation metrics: Fusion image performance is mainly divided into two categories: subjective evaluation and objective evaluation. Subjective evaluation is generally based on the visual perception of the user. Usually, the more infrared salient targets and visible detailed textures contained in the fusion image there are, the better the user’s subjective evaluation is. Objective evaluations typically use suitable quantitative metrics to assess the performance of the fusion network. In this paper, the following six objective metrics were selected: entropy (EN) [30], standard deviation (SD) [31], mutual information content (MI) [32], structural similarity measure (SSIM) [29], visual fidelity (VIF) [33], and sum of difference correlation (SCD) [34].

The mathematical formula of EN, which is a measurement of the amount of information in an image, is as follows:

(11) $$EN=-\sum_{l=0}^{L-1}p_{l}{log}_{2}p_{l}$$

where *L* is the total number of gray levels and $p_{l}$ is the average distribution of matching gray-level fusion images. Larger EN indicates more information contained in the fusion image and better performance of the network.

MI is a metric that quantifies how much information has passed from the source to the fusion image. It mainly computes the correlation between source and fusion images. The MI mathematical formula is as follows:

(12) $$MI_{X,F}=\sum_{x,f}P_{X,F}\left(x,f\right)log\frac{P_{X,F}\left(x,f\right)}{P_{X}\left(x\right)P_{F}\left(f\right)}$$

where $P_{X,F}\left(x,f\right)$ is the joint histogram of the source and fusion images, and $P_{X}\left(x\right)$ and $P_{F}\left(f\right)$ are the edge histograms of the source and fusion images. The higher MI is, the more information of the source image the fusion image contains.

SD is a metric of a fused image’s contrast and distribution. The SD mathematical formula is as follows:

(13) $$SD=\sqrt{\sum_{i=1}^{M}\sum_{j=1}^{N}\left({F\left(i,j\right)-\mu )}^{2}\right.}$$

where mu is the average of the fusion image pixels. Because the human visual system pays a lot of attention to high-contrast regions, fusion results with higher SD have better contrast.

SSIM is a metric that measures the structural similarity between the fusion image and the source image, and its formula is shown in Equation (3); its range is [−1, 1]. When SSIM is equal to 1, the two images are identical. Therefore, the higher the index is, the worse it is. If SSIM is too high, it reflects that the fusion images are too similar to the source images and lack creativity and distinctiveness.

VIF is a metric that quantifies the amount of information shared by the fusion and source images using the human visual system and natural scene statistics. The VIF mathematical formula is as follows:

(14) $$VIF_{X,F}=\frac{\sum_{i\in subbands}\left.I\left({\vec{C}}^{S,i},{\vec{F}}^{S,i}\;\right|R^{S,i}\;\right)}{\sum_{i\in subbands}\left.I\left({\vec{C}}^{S,i},{\vec{X}}^{S,i}\;\right|R^{S,i}\;\right)}$$

SCD is a metric for assessing the information richness within a fusion image that measures the difference between the source and fusion images. The SCD mathematical formula is as follows:

(15) $$SCD_{X,F}=\frac{\sum_{i}\sum_{j}\left(X-\bar{X}\right)\left(F-\bar{F}\right)}{\sqrt{\sum_{i}\sum_{j}{\left(X-\bar{X}\right)}^{2}\sum_{i}\sum_{j}{\left(F-\bar{F}\right)}^{2}}}$$

RSDFusion was compared with nine of today’s most popular methods: two traditional algorithms, namely, MDLatLRR [11] and GTF [35]; three AE-based methods, namely, DenseFuse [14], NestFuse [15], and RFN-Nest [36]; two CNN-based algorithms, namely, IFCNN [17] and SeAFusion [18]; two GAN-based algorithms, namely, FusionGAN [19] and U2Fusion [37]. Test images were obtained from the above nine publicly available image fusion algorithms.

(3) Training setup: We used the RoadScene dataset to train our RSDFusion model. From it, 180 pairs of infrared and visible images were selected; then, a semantic segmentation network was used to segment these images. Each set of images after segmentation contained an infrared image, an infrared semantic image, a visible image, and a visible semantic image. A sliding window of 256 × 384 with a step size of 32 was used to crop the images to obtain more training images. After cropping, a total of 1908 image sets were obtained for training. In the test, 21 pairs of typical images were chosen from the TNO and RoadScene datasets for comparison experiments. In addition, the hyperparameters in network training were defined as follows: the training batch size was 16; the number of iterations was 10; the learning rate was 5 × 10${}^{-4}$; and the optimizer was Adam. In addition, the proposed technique was built on the PyTorch platform. All experiments were performed on Intel i9-11900 and NVIDIA GeForce RTX 3090.

### 4.2. Comparative Experiments

We compared RSDFusion with nine other methods on the RoadScene dataset to evaluate our performance.

**Figure 4:**
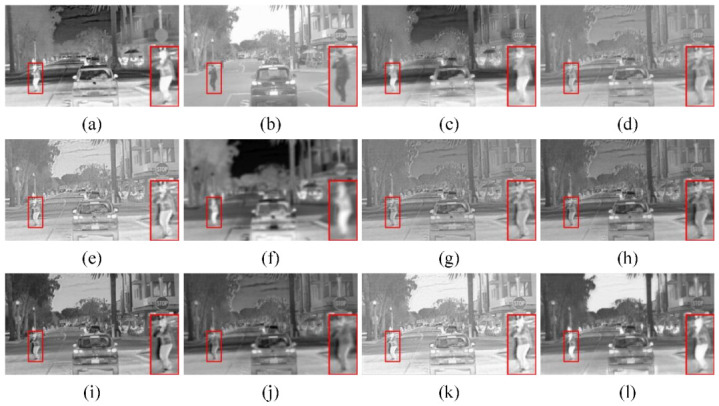
Qualitative comparison of RSDFusion and nine state-of-the-art methods on image FLIR_08749. (**a**) Infrared image, (**b**) visible image, (**c**) GTF, (**d**) MDLatLRR, (**e**) IFCNN, (**f**) FusionGAN, (**g**) U2Fusion, (**h**) DenseFuse, (**i**) NestFuse, (**j**) RFN-Nest, (**k**) SeAFusion, and (**l**) RSDFusion.

**Figure 5:**
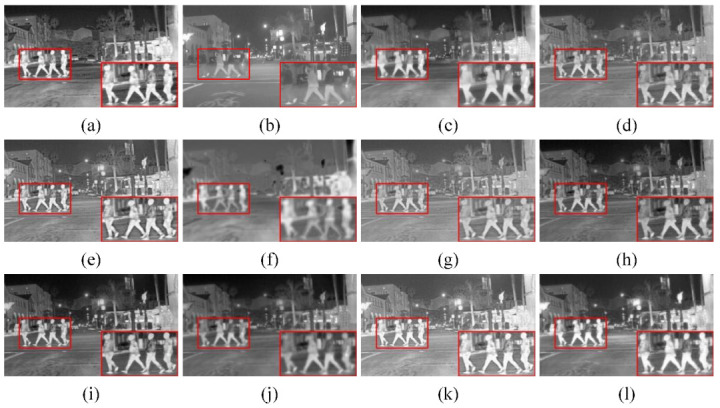
Qualitative comparison of RSDFusion and nine state-of-the-art methods on image FLIR_08874. (**a**) Infrared image, (**b**) visible image, (**c**) GTF, (**d**) MDLatLRR, (**e**) IFCNN, (**f**) FusionGAN, (**g**) U2Fusion, (**h**) DenseFuse, (**i**) NestFuse, (**j**) RFN-Nest, (**k**) SeAFusion, and (**l**) RSDFusion.

(1) Qualitative comparison: To visually compare the performance difference between our method and other algorithms, we selected two pairs of typical source images from the RoadScene dataset for subjective evaluation, both of which contained semantic segmentation objects to be extracted and focused on in subsequent evaluations and which were captured in the daytime and nighttime, respectively. The results of the compared algorithms are shown in Figure 4 and Figure 5. In the figures above, we selected a salient object (i.e., red box) from each set of fusion images and enlarged it in the lower right corner of the fusion image for observation. As shown in Figure 4, the infrared target was severely missing with U2Fusion and could not be captured significantly. There was a lack of background information with FusionGAN and GTF, such as the lack of detailed texture information of clouds in the sky. The RFN-Nest fusion image had low contrast, and the overall brightness was dark. The infrared target information was retained with IFCNN and MDLatLRR, but the target saliency was insufficient. In contrast, SeAFusion and RSDFusion could retain high-quality infrared targets. In particular, RSDFusion provided more detailed texture information while highlighting the infrared target, which is more realistic in terms of visual effects.

As shown in Figure 4, RSDFusion retained high-quality infrared targets and relatively more visible detailed texture information. Only SeAFusion and our method could observe the mountains and trees in the background, but the contrast between the main targets and the background was higher in RSDFusion fusion images. The above qualitative comparison shows that RSDFusion can extract high-quality targets while preserving rich detailed texture information. The targets extracted with the semantic network mechanism better preserve the target information and edge area of the source image, thus producing images with more comfortable and good subjective visual perception.

**Figure 6:**
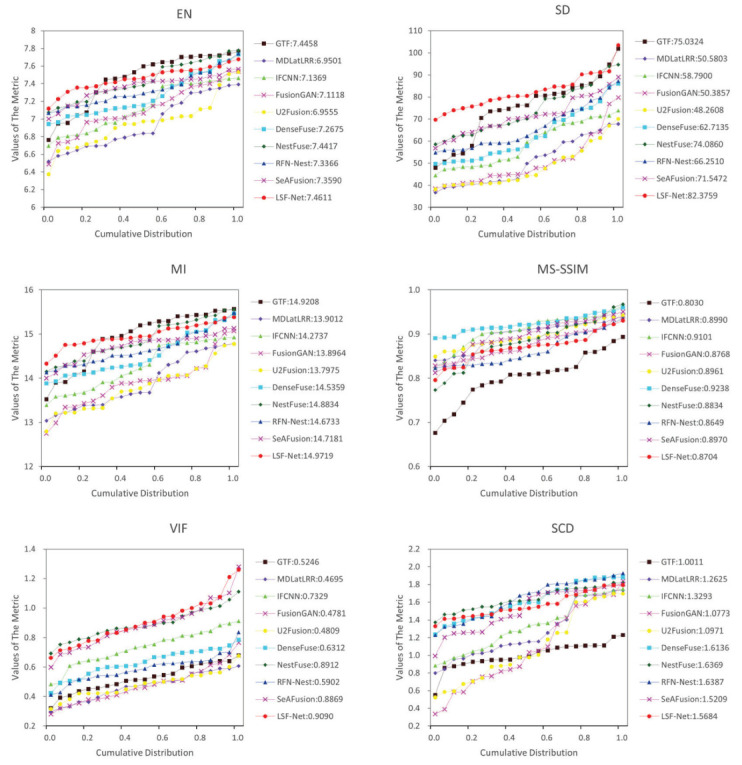
Twenty-one pairs of images from the RoadScene dataset were compared quantitatively on six metrics, namely, EN, SD, MI, SSIM, VIF, and SCD. The point on the curve (x, y) indicates that there are (100 × x)% of image pairs with metric values not exceeding y.

(2) Quantitative comparison: A total of 21 pairs of images from the RoadScene dataset were selected for quantitative comparison. The objective comparison results of each method on six metrics are shown in Figure 6 and Table 1. On the RoadScene dataset, RSDFusion achieved the best results, with significant advantages in four metrics, EN, SD, MI, and VIF, and the rest of the metrics were at an average level. Among them, the highest value of EN indicates that the fusion images generated using RSDFusion were richer in information. The highest value of VIF indicates that the fusion image had the best visual effect. The value of MS-SSIM indicates that the fusion image generated using RSDFusion had higher brightness and contrast than the source image, which reduces the similarity of brightness and contrast in this index.

In summary, extensive experimental controls on the RoadScene dataset show that our algorithm has superior semantic target object extraction and detailed texture retention, and the generated fusion images have superior human visual effects. We attribute this to the following factors: First, we accurately capture the extracted targets using semantic loss, which improves the network’s control over local images. Second, we constrain the fusion image to retain more detailed texture information of the source image while ensuring the extraction of the focal target using the global structural loss function.

### 4.3. Generalization Comparison

To evaluate the generalization performance of the model, further comparisons were performed on the TNO dataset.

**Figure 7:**
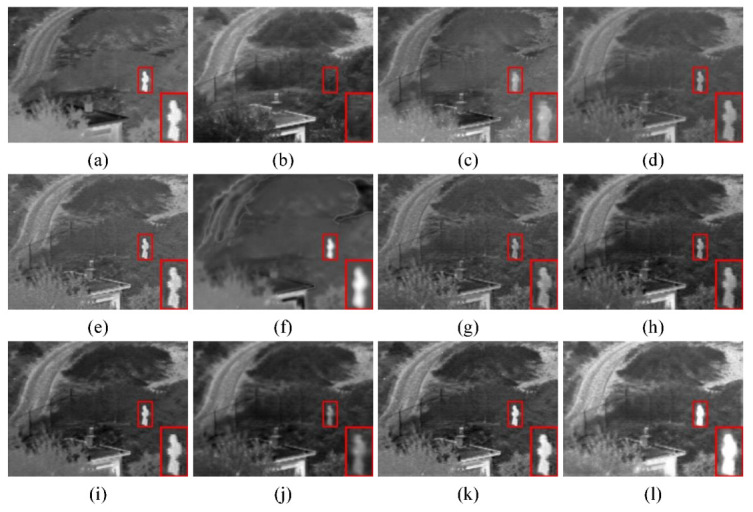
Qualitative comparison of RSDFusion and nine state-of-the-art methods on image Nato_camp. (**a**) Infrared image, (**b**) visible image, (**c**) GTF, (**d**) MDLatLRR, (**e**) IFCNN, (**f**) FusionGAN, (**g**) U2Fusion, (**h**) DenseFuse, (**i**) NestFuse, (**j**) RFN-Nest, (**k**) SeAFusion, and (**l**) RSDFusion.

**Figure 8:**
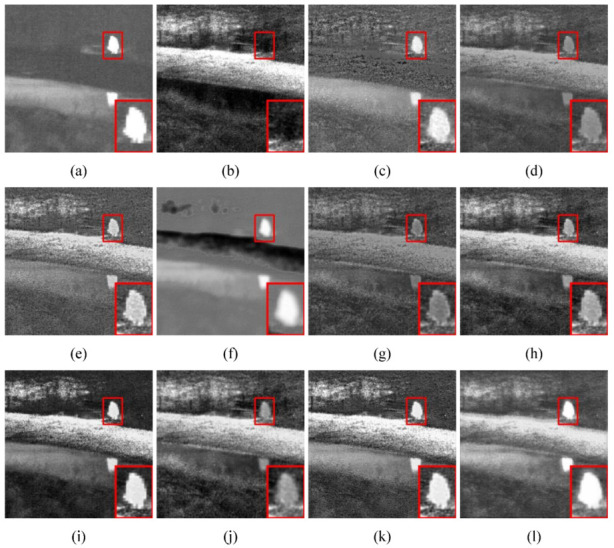
Qualitative comparison of RSDFusion and nine state-of-the-art methods on image Bench. (**a**) Infrared image, (**b**) visible image, (**c**) GTF, (**d**) MDLatLRR, (**e**) IFCNN, (**f**) FusionGAN, (**g**) U2Fusion, (**h**) DenseFuse, (**i**) NestFuse, (**j**) RFN-Nest, (**k**) SeAFusion, and (**l**) RSDFusion.

**Figure 9:**
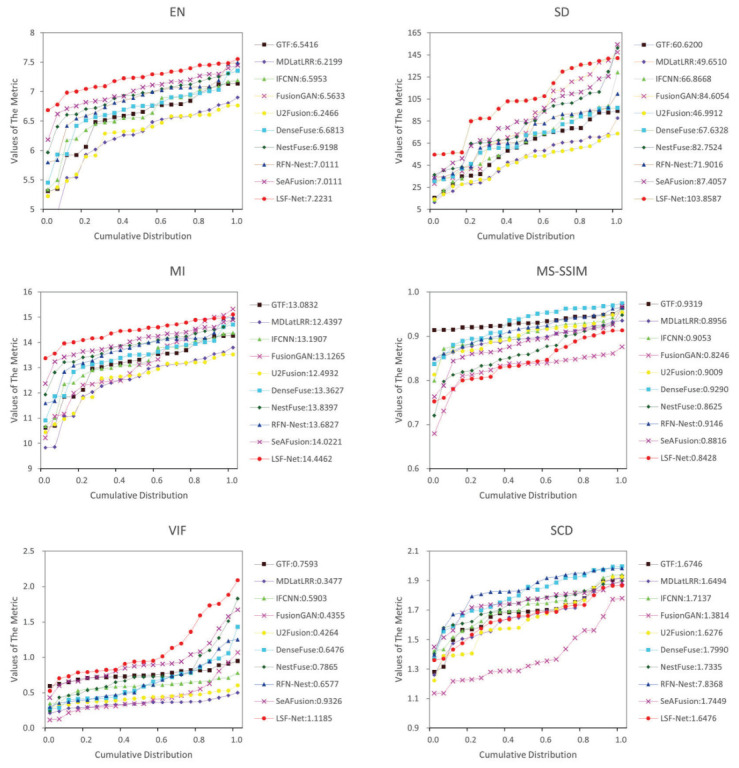
Twenty-one pairs of images from the TNO dataset were compared quantitatively on six metrics, namely, EN, SD, MI, SSIM, VIF, and SCD. The point on the curve (x, y) indicates that there are (100 × x)% of image pairs with metric values not exceeding y.

(1) Qualitative comparison: Two pairs of typical source images were selected from the TNO dataset for subjective evaluation; they were taken in clear and foggy scenes at night, respectively. Their comparison results are shown in Figure 7 and Figure 8. First, as shown in Figure 7, the infrared target was severely lost with RFN-Nest, and no infrared target could be significantly detected. Second, the brightness of the targets detected with MDLatLRR and DenseFuse was too large compared with the targets in the infrared images, and the edge positions were blurred. In addition, infrared target information was retained with GTF, IFCNN, and NestFuse, but there was information contamination of infrared targets by visible images. U2Fusion, STDFusion, SeAFusion, and RSDFusion could retain high-quality infrared targets. In particular, RSDFusion had more detailed texture information while highlighting the infrared target, which is more realistic in visual effect. According to Figure 8, RSDFusion is the only method that preserved both the visible detail features on the right and the infrared flow targets.

(2) Quantitative comparison: A total of 21 pairs of images from the TNO dataset were selected for quantitative comparison. The objective evaluation results are shown in Figure 9 and Table 2. Similar to the metrics in the RoadScene dataset, RSDFusion achieved the best results, with significant advantages in EN, SD, MI, and VIF metrics on the TNO dataset, and the advantages were more obvious compared with the RoadScene dataset, which was due to the greater brightness and contrast between the main target and the background of the source images in the TNO dataset. In addition, our algorithm still had the best stability on the TNO dataset. Overall, qualitative and quantitative experiments show that RSDFusion has a good generalization ability.

### 4.4. Efficiency Comparison

In image fusion tasks, operational efficiency is a significant factor in evaluating the performance of image fusion models. In this paper, the designed fusion model, RSDFusion, is a lightweight real-time network that must have efficient operational efficiency to support real-time image fusion. The efficiency of RSDFusion and nine other methods was tested on the RoadScene and TNO datasets in the same hardware environment. As shown in Table 3, all methods using deep learning had significant advantages in terms of operational efficiency. In particular, our method outperformed all other methods except IFCNN on the TNO and RoadScene datasets. Overall, RSDFusion has short and stable average running time, which enables real-time image fusion.

### 4.5. Ablation Experiments

**Figure 10:**
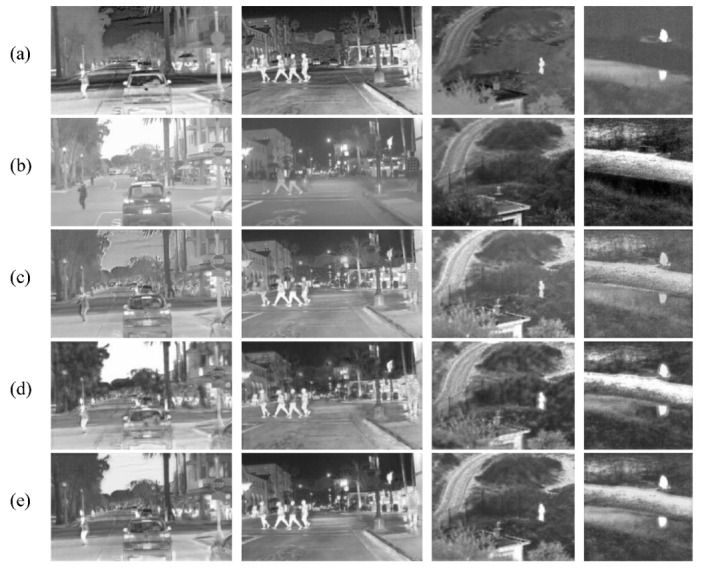
Ablation experiments were performed on four typical image pairs in the test set. From top to bottom: (**a**) infrared image, (**b**) visible image, (**c**) image without semantic loss, (**d**) image without SSIMLoss, and (**e**) RSDFusion image.

In our model, semantic loss and structural loss are significant components of the training network. To verify the soundness of the designed semantic and structural loss functions, we trained the fusion model without semantic and structural loss, respectively. As shown in Figure 10, if trained without semantic loss, the network unintentionally retains the most significant information of the source image, resulting in poor distinction between the main semantic target and the background of the final fusion image. In the case of training without structural loss guidance, the quality of the fusion image is low, with problems such as blurred target edges and a lack of detailed texture information. In contrast, the fusion result of RSDFusion preserves not only the semantic targets of the source image but also the rich texture details of the source image.

In Table 4, our RSDFusion is the best according to the four metrics of EN, SD, MI, and VIF. Thus, our method can retain clearer and richer semantic targets, and superior human visuals.

## 5. Conclusions

In this paper, we propose a new real-time semantics-driven infrared and visible image fusion framework, RSDFusion. The framework introduces semantic segmentation into image fusion, divides the source image into several semantic objects, designs an adapted loss function for its important semantic target objects, and combines it with global structural loss to drive network training. As a result, the fusion images we generate not only preserve the important targets of the source images but also retain a large amount of the texture detail information of the source images. The method also has the limitations that the generality of the task needs to be improved and the fusion network needs to be retrained when the target of interest for the task changes. In extensive qualitative and quantitative experiments on the RoadScene and TNO datasets, our fusion method RSDFusion outperformed other popular methods in both subjective visualization and objective metric measures. Furthermore, the efficient operation of our algorithm supports the real-time fusion of source images.

## Figures and Tables

**Table 1:** Twenty-one pairs of images from the RoadScene dataset were compared quantitatively on six metrics, namely, EN, SD, MI, SSIM, VIF, and SCD. The red color represents the best result, and the blue color represents the second-best result.

	EN	SD	MI	MS-SSIM	VIF	SCD
GTF [35]	7.4558	73.0327	14.9208	0.8030	0.5246	1.0011
MDLatLRR [11]	6.9506	50.5803	13.9012	0.8990	0.4695	1.2325
IFCNN [17]	7.1369	58.7900	14.2737	0.9101	0.7329	1.3293
FusionGAN [19]	7.1118	50.3857	13.8964	0.8768	0.4781	1.0773
U2Fusion [37]	6.9555	48.2608	13.7975	0.8961	0.4809	1.0971
DenseFuse [14]	7.2675	62.7135	14.5349	0.9238	0.6312	1.6136
NestFuse [15]	7.4417	74.0860	14.8834	0.8834	0.8916	1.6369
RFN-Nest [36]	7.3366	66.2510	14.6733	0.8649	0.5902	1.6387
SeAFusion [18]	7.3590	71.5472	14.7181	0.8970	0.8869	1.5209
RSDFusion (Ours)	7.4611	82.3759	14.9719	0.8704	0.9090	1.5684

**Table 2:** Twenty-one pairs of images from the TNO dataset were compared quantitatively on six metrics, namely, EN, SD, MI, SSIM, VIF, and SCD. The red color represents the best result, and the blue color represents the second-best result.

	EN	SD	MI	MS-SSIM	VIF	SCD
GTF [35]	6.5416	90.9200	13.0832	0.9319	0.7593	1.6746
MDLatLRR [11]	6.2199	49.6511	12.4397	0.8956	0.3477	1.6494
IFCNN [17]	6.5953	66.8668	13.1907	0.9053	0.5903	1.7137
FusionGAN [19]	6.5633	84.6054	13.1265	0.8246	0.4355	1.3814
U2Fusion [37]	6.2466	46.9912	12.4932	0.9009	0.4264	1.6276
DenseFuse [14]	6.6813	67.6328	13.3627	0.9290	0.6476	1.7990
NestFuse [15]	6.9198	82.7524	13.8397	0.8625	0.7895	1.7335
RFN-Nest [36]	6.8414	71.9016	13.6827	0.9146	0.6577	1.8368
SeAFusion [18]	7.0111	87.4057	14.0221	0.8816	0.9326	1.7449
RSDFusion (Ours)	7.2231	103.8587	14.4462	0.8428	1.1158	1.6476

**Table 3:** Time means and standard deviations of running RSDFusion and the remaining nine methods on the RoadScene and TNO datasets, with red indicating the best results and blue, the second-best results.

	RoadScene	TNO
GTF [35]	0.8749 + 1.0116	2.3931 + 1.3194
MDLatLRR [11]	20.1372 + 4.2147	40.9576 + 13.8720
IFCNN [17]	0.0077 + 0.0030	0.0149 + 0.0078
FusionGAN [19]	1.3039 + 0.2174	1.4587 + 0.3558
U2Fusion [37]	0.5178 + 0.1064	1.0105 + 0.4211
DenseFuse [14]	0.2139 + 0.0254	0.3629 + 0.1102
NestFuse [15]	0.1778 + 0.4340	0.2915 + 0.4584
RFN-Nest [36]	0.3153 + 0.0685	0.3445 + 0.4505
SeAFusion [18]	0.0652 + 0.2783	0.0863 + 0.3041
RSDFusion	0.0604 + 0.2514	0.0672 + 0.2424

**Table 4:** In a comparison of the ablation experiments performed on 21 images from the RoadScene dataset, six metrics were used: EN, MI, VIF, SD, AG, and SF.

	EN	SD	MI	SSIM	VIF	SCD
W/o semantic	7.2876	65.4618	14.5753	0.8916	0.7518	1.6608
W/o SSIMLoss	7.3703	81.4396	14.7407	0.7464	0.7799	1.3501
RSDFusion	7.4611	82.3759	14.9719	0.8704	0.9090	1.5684

## Data Availability

Not applicable.

## References

[1] Zhang H., Xu H., Tian X., Jiang J., Ma J. (2021). Image fusion meets deep learning: A survey and perspective. Inf. Fusion.

[2] Ma J., Ma Y., Li C. (2019). Infrared and visible image fusion methods and applications: A survey. Inf. Fusion.

[3] Cao Y., Guan D., Huang W., Yang J., Cao Y., Qiao Y. (2019). Pedestrian detection with unsupervised multispectral feature learning using deep neural networks. Inf. Fusion.

[4] Bhatnagar G., Wu Q.J., Liu Z. (2013). Directive contrast based multimodal medical image fusion in NSCT domain. IEEE Trans. Multimed..

[5] Ha Q., Watanabe K., Karasawa T., Ushiku Y., Harada T. MFNet: Towards real-time semantic segmentation for autonomous vehicles with multi-spectral scenes. Proceedings of the 2017 IEEE/RSJ International Conference on Intelligent Robots and Systems (IROS).

[6] Simone G., Farina A., Morabito F.C., Serpico S.B., Bruzzone L. (2002). Image fusion techniques for remote sensing applications. Inf. Fusion.

[7] Ben Hamza A., He Y., Krim H., Willsky A. (2005). A multiscale approach to pixel-level image fusion. Integr. Comput.-Aided Eng..

[8] Chen J., Li X., Luo L., Mei X., Ma J. (2020). Infrared and visible image fusion based on target-enhanced multiscale transform decomposition. Inf. Sci..

[9] Wang J., Lu C., Wang M., Li P., Yan S., Hu X. (2014). Robust face recognition via adaptive sparse representation. IEEE Trans. Cybern..

[10] Liu Y., Chen X., Ward R.K., Wang Z.J. (2016). Image fusion with convolutional sparse representation. IEEE Signal Process. Lett..

[11] Li H., Wu X.J., Kittler J. (2020). MDLatLRR: A novel decomposition method for infrared and visible image fusion. IEEE Trans. Image Process..

[12] Cvejic N., Bull D., Canagarajah N. (2007). Region-based multimodal image fusion using ICA bases. IEEE Sens. J..

[13] Ma J., Zhou Z., Wang B., Zong H. (2017). Infrared and visible image fusion based on visual saliency map and weighted least square optimization. Infrared Phys. Technol..

[14] Li H., Wu X.J. (2018). DenseFuse: A fusion approach to infrared and visible images. IEEE Trans. Image Process..

[15] Li H., Wu X.J., Durrani T. (2020). NestFuse: An infrared and visible image fusion architecture based on nest connection and spatial/channel attention models. IEEE Trans. Instrum. Meas..

[16] Liu Y., Chen X., Cheng J., Peng H., Wang Z. (2018). Infrared and visible image fusion with convolutional neural networks. Int. J. Wavelets Multiresolut. Inf. Process..

[17] Zhang Y., Liu Y., Sun P., Yan H., Zhao X., Zhang L. (2020). IFCNN: A general image fusion framework based on convolutional neural network. Inf. Fusion.

[18] Tang L., Yuan J., Ma J. (2022). Image fusion in the loop of high-level vision tasks: A semantic-aware real-time infrared and visible image fusion network. Inf. Fusion.

[19] Ma J., Yu W., Liang P., Li C., Jiang J. (2019). FusionGAN: A generative adversarial network for infrared and visible image fusion. Inf. Fusion.

[20] Ma J., Xu H., Jiang J., Mei X., Zhang X.P. (2020). DDcGAN: A dual-discriminator conditional generative adversarial network for multi-resolution image fusion. IEEE Trans. Image Process..

[21] Li J., Huo H., Li C., Wang R., Feng Q. (2020). AttentionFGAN: Infrared and visible image fusion using attention-based generative adversarial networks. IEEE Trans. Multimed..

[22] Long J., Shelhamer E., Darrell T. Fully convolutional networks for semantic segmentation. Proceedings of the IEEE Conference on Computer Vision and Pattern Recognition.

[23] Ronneberger O., Fischer P., Brox T. (2015). U-net: Convolutional networks for biomedical image segmentation. Lecture Notes in Computer Science, Proceedings of the Medical Image Computing and Computer-Assisted Intervention–MICCAI 2015: 18th International Conference, Munich, Germany, 5–9 October 2015.

[24] Chen L.C., Papandreou G., Kokkinos I., Murphy K., Yuille A.L. (2017). Deeplab: Semantic image segmentation with deep convolutional nets, atrous convolution, and fully connected crfs. IEEE Trans. Pattern Anal. Mach. Intell..

[25] Xie E., Wang W., Yu Z., Anandkumar A., Alvarez J.M., Luo P. (2021). SegFormer: Simple and efficient design for semantic segmentation with transformers. Adv. Neural Inf. Process. Syst..

[26] Hou J., Zhang D., Wu W., Ma J., Zhou H. (2021). A generative adversarial network for infrared and visible image fusion based on semantic segmentation. Entropy.

[27] Ciocca G., Napoletano P., Schettini R. (2018). CNN-based features for retrieval and classification of food images. Comput. Vis. Image Underst..

[28] Zhou B., Zhao H., Puig X., Fidler S., Barriuso A., Torralba A. Scene parsing through ade20k dataset. Proceedings of the IEEE Conference on Computer Vision and Pattern Recognition.

[29] Ma K., Zeng K., Wang Z. (2015). Perceptual quality assessment for multi-exposure image fusion. IEEE Trans. Image Process..

[30] Roberts J.W., Van Aardt J.A., Ahmed F.B. (2008). Assessment of image fusion procedures using entropy, image quality, and multispectral classification. J. Appl. Remote Sens..

[31] Rao Y.J. (1997). In-fibre Bragg grating sensors. Meas. Sci. Technol..

[32] Qu G., Zhang D., Yan P. (2002). Information measure for performance of image fusion. Electron. Lett..

[33] Han Y., Cai Y., Cao Y., Xu X. (2013). A new image fusion performance metric based on visual information fidelity. Inf. Fusion.

[34] Aslantas V., Bendes E. (2015). A new image quality metric for image fusion: The sum of the correlations of differences. Aeu-Int. J. Electron. Commun..

[35] Ma J., Chen C., Li C., Huang J. (2016). Infrared and visible image fusion via gradient transfer and total variation minimization. Inf. Fusion.

[36] Li H., Wu X.J., Kittler J. (2021). RFN-Nest: An end-to-end residual fusion network for infrared and visible images. Inf. Fusion.

[37] Xu H., Ma J., Jiang J., Guo X., Ling H. (2020). U2Fusion: A unified unsupervised image fusion network. IEEE Trans. Pattern Anal. Mach. Intell..

